# Exploring biomarkers associated with severity of knee osteoarthritis in Southern China using widely targeted metabolomics

**DOI:** 10.1186/s12891-023-07084-4

**Published:** 2023-12-08

**Authors:** Xiaochao Wang, Wanling Cai, Yihan Liu, Yaoming Lu, Mange Liu, Xuewei Cao, Da Guo

**Affiliations:** 1 Guangzhou University of Chinese Medicine, Guangzhou, Guangdong China; 2 Shuguang Hospital Affiliated to Shanghai University of Chinese Traditional Medicine, Shanghai, China; 3 Department of Neurological Rehabilitation, The Second Affiliated Hospital of Zhengzhou University, Zhengzhou, China; 4 Department of Orthopaedic Surgery, Guangdong Provincial Hospital of Chinese Medicine, Guangzhou, Guangdong China

**Keywords:** Knee osteoarthritis, Metabolomics, Biomarkers

## Abstract

**Background:**

Metabolomics is a tool to study the pathogenesis of diseases and their associated metabolites, but there are still insufficient metabolomic studies on severe knee osteoarthritis.To investigate the differences in serum metabolites between healthy populations and knee osteoarthritis (KOA) patients in Southern China using widely targeted metabolomics, and to explore biomarkers and their metabolic pathways that could be associated with the severity of KOA.

**Methods:**

There were 10 healthy individuals in the control group and 32 patients with KOA. According to the Kellgren–Lawrence (KL) grading system, KOA was further divided into mild (n = 13, KL grade 1 and 2) and severe (n = 19, KL grade 3 and 4). Serum samples from all participants were collected and analyzed metabolomics based on ultra-performance liquid chromatography/electrospray ionization/tandem mass spectrometry. We screened for differential metabolites between patients and controls, and between mild and severe KOA. We explored the metabolic pathways involved in differential metabolism using the Kyoto Encyclopedia of Genes and Genomes database.

**Results:**

Sixty-one metabolites were differentially expressed in the sera of the patient group compared with the control group (45 upregulated and 16 downregulated). Analysis of the mild and severe KOA groups showed a total of 12 differential metabolites. Receiver operating characteristic curve analysis showed N-alpha-acetyl-L-asparagine was a good predictor of advanced osteoarthritis(OA).Differential metabolites are enriched in multiple pathways such as arachidonic acid metabolism.

**Conclusion:**

Widely targeted metabolomics found that upregulation of the amino acid metabolite N-α-acetyl-L-asparagine was significantly associated with severe KOA and could be a biomarker for predicting severity of KOA. Arachidonic acid metabolism may play an important role in patients with severe KOA.

## Background

Osteoarthritis (OA) is an inflammatory disease of the synovial joints [1, 2]. It is the most frequent form of arthritis and a major contributor to pain and disability in older people [3, 4]. Knee OA (KOA) is common [5], and with an increasingly aging population, > 250 million people worldwide have KOA [6]. The pain and financial burden are the main effects of KOA on both the individual and society [7, 8]. Early prevention and timely diagnosis are important aspects of disease management, but the detailed etiology, pathophysiology and metabolic mechanisms are not yet fully understood [9]. The diagnosis of KOA currently relies on imaging techniques, and X-ray analysis is still the most widely used examination [10]. However, when there are subtle pathological changes within the joint, they cannot be identified on by X-ray [11]. Therefore, new methods for evaluation of KOA are being sought.

Metabolomics is a new discipline developed after genomics, transcriptomics and proteomics [12].Metabolomics can analyze the small molecules of endogenous metabolites in organisms and shed light on biological change [13].Widely targeted metabolomics is one of the metabolomics technologies, which combines the advantages of targeted and non-targeted metabolomics, and is more suitable for the detection of low to medium abundance metabolites [14, 15].Recent research has focused on the biomarkers and pathophysiology of OA using metabolomics [16]. Metabolomics studies consist of three main steps: sample preparation, metabolome assay, and data analysis, through which small molecule metabolites in tissues can be identified [17]. The application of metabolomics to the study of KOA has identified several biomarkers associated with KOA [18–20]. Researchers have also begun to investigate the correlation between biomarkers and the severity of KOA [3, 21, 22]. Although different metabolites were obtained in these studies due to different sample sources, these findings have led to a better understanding of the pathogenesis of KOA. In metabolomics studies of KOA, peripheral blood samples and synovial fluid are the two most commonly used sources. Collection of synovial fluid is an invasive procedure and involves some risk [23]. Blood samples have ease of access and detection of overall metabolic characteristics, making them the first choice for most studies [16].

Currently,The pathogenesis of knee osteoarthritis remains incompletely understood and the biomarkers associated with severe knee osteoarthritis remain unclear.Therefore, this study explored the metabolite differences between KOA patients and healthy individuals in Southern China using widely targeted metabolomics, and aimed to identify biomarkers that could differentiate KOA severity.

## Materials and methods

### Participants

The inclusion criteria for patients were: diagnosis of primary osteoarthritis of the knee, age between 50 and 82 years, and no use of nonsteroidal anti-inflammatory drugs or hormonal drugs in the last week. Finally,thirty-two KOA patients were recruited from the Orthopedic Department of Guangdong Provincial Hospital of Chinese Medicine between Jan 2019 and Aug 2019. According to Kellgren–Lawrence (KL) grading system [24], KOA was further divided into mild (n = 13, KL grade 1 and 2) and severe (n = 19,KL grade 3 and 4) disease. The diagnostic criteria for KOA were based on the American College of Rheumatology Clinical symptomatic and radiographic criteria [25]. Patients with rheumatoid arthritis or secondary OA, autoimmune disease, malignancy, systemic inflammatory or infectious disease and severe liver or kidney disease were excluded. During the same time period, 10 healthy individuals were recruited from the clinical department of GuangdongProvincial Hospital of Traditional Chinese Medicine who were matched for age and gender to the KOA group. Healthy participants were excluded if they had OA-related symptoms and imaging changes, autoimmune disease, malignancy, rheumatoid arthritis, advanced liver and kidney disease, and infectious disease within the past 3 months. None of the enrolled healthy volunteers had a history of musculoskeletal disease or knee injury.Demographic information such as gender, age and body mass index (BMI) was recorded for both groups.There were five participants in the patient group and two in the control group with a BMI greater than 30. There were no participants with a BMI of less than 18.5 in either group. Written informed consent was obtained from all participants prior to the start of the study. The study was conducted in accordance with the ethical standards of the 1964 Declaration of Helsinki and was approved by the Ethics Committee of Guangdong Provincial Hospital of Chinese Medicine(approval number: Z2017-110).

### Preparation of serum samples

All patients were fasted for at least 8 h before collecting blood samples. Blood samples were centrifuged for 15 min (1500 g) and serum was extracted and stored at −80 °C until analysis was performed. The samples were removed from the freezer and thawed on ice until the samples were completely free of ice. After thawing, the samples were vortexed for 10 s, and 50-µL samples were removed and added to an empty centrifuge tube with 300 µL of internal standard extract. The mixture was vortexed and mixed, and centrifuged at 12,000 g for 10 min at 4 °C. We collected 200 µL of the supernatant in a new tube and centrifuged again at 12,000 g for 3 min at 4 °C. After centrifugation, 150 µL of the extracted supernatant was added to the liner tube of the injection vial and left for analysis.

### Metabolome analysis

Samples were analyzed using an ExionLC AD ultra-performance liquid chromatography (UPLC) system combined with a QTRAP® tandem mass spectrometry (MS) system (Applied Biosystems, Foster City, CA, USA). Sample extract (2 µL) was injected onto a Waters Acquity UPLC HSS T3 C18 (1.8 μm, 2.1 and 100 mm) column. The column temperature was set at 40 °C and the flow rate was 0.4 mL/min. The mobile phase consisted of ultrapure water (0.1% formic acid) in phase A and acetonitrile (0.1% formic acid) in phase B. The elution gradients were specified as follows: 0 min 95:5 V/V, 10.0 min 10:90 V/V, 11.0 min 10:90 V/V, 11.1 min 95:5 V and 14.0 min 95:5 V/V. Mass measurements were carried out in positive and negative ion electrospray mode. Triple quadrupole MS was used. The software Analyst 1.6.3 (Sciex) was used to process the MS data. The electrospray ion source parameters were as follows: source temperature 500 °C, MS voltage set to 5500 V (positive) and −4500 V (negative); ion source gas I, gas II and curtain gas set to 55, 60 and 25 psi respectively; collision gas set to high. A quality control sample was inserted in every 10 samples during the analysis to monitor reproducibility.

### Statistical analysis

SPSS 24.0 statistical software package was used to analyze the data. For the demographic and laboratory data of both groups, quantitative information was described as mean±standard deviation. For quantitative information, independent-samples Student’s t test was chosen for the assessment between the two groups. Correlations between categorical variables were assessed using the χ^2^ test or Fisher’s exact test. The results were considered statistically significant at *P* ≤ 0.05.

The data analysis and statistical methods for metabolites were as follows. The control and patient groups were analyzed using the orthogonal partial least square-discriminate analysis (OPLS-DA). Variable importance in projection (VIP) combined with the fold change was used to screen for differential metabolites. Metabolites with fold change ≥ 2 and fold change ≤ 0.5 were selected in the first step, and metabolites with VIP ≥ 1 were selected if there was biological duplication in the sample grouping on the basis of the above. The same method was used to analyze the mild and severe KOA groups to obtain the differential metabolites. Receiver operating characteristic (ROC) curve analysis was performed to examine the predictive ability of biomarkers to distinguish between patients with mild and severe KOA by calculating the area under the curve (AUC). The obtained differential metabolites were analyzed using the Kyoto Encyclopedia of Genes and Genomes (KEGG) pathway for enrichment.

## Results

### Participants

There were 32 KOA patients, including 7 men and 25 women with a mean age of 66 ± 5.51 years, and 10 healthy individuals, including 2 men and 8 women, aged 68.9 ± 4.28 years. There were no significant differences between KOA patients and healthy controls in terms of age, sex and BMI (*P* > 0.05 for all parameters). The demographic characteristics of the two groups are shown in Table 1.

**Table 1:** Comparison of demographic characteristics of patient and control groups

	patient group (n = 32)	control group (n = 10)	*p*-value
Age(years)	68.13 ± 6.24	68.90 ± 4.28	0.717
Gender(Women %)	78.10%	80.00%	1.000
BMI(kg/m2)	27.09 ± 3.62	27.49 ± 2.85	0.754

Further statistical analysis of the demographic characteristics (age, gender and BMI) of patients in the mild versus severe KOA groups was performed (Table 2). None of the parameters were significantly different (*P* > 0.05).

**Table 2:** Comparison of demographic characteristics of mild and severe KOA groups

	mild group (n = 13)	severe group (n = 19)	*p*-value
Age(years)	66 ± 5.51	69.58 ± 6.43	0.112
Gender(Women %)	76.90%	78.90%	1.000
BMI(kg/m2)	26.01 ± 2.15	27.84 ± 4.25	0.121

### Identification of metabolites

**Fig. 1:**
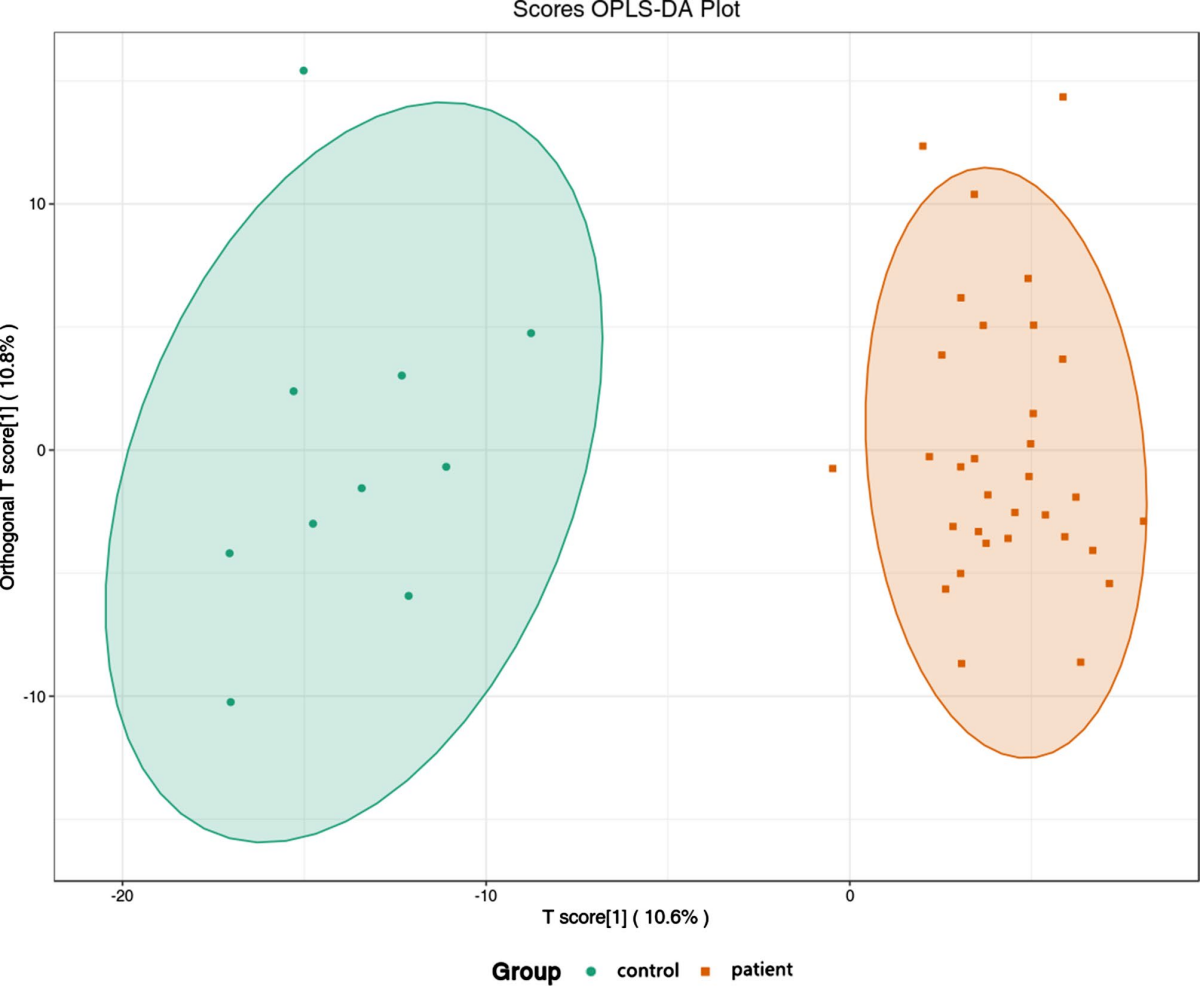
Plot of OPLS-DA scores in the control versus patient groups. One circle represents one participant in the control group, one square represents one patient in the patient group. The samples of both groups showed the trend of intra-group aggregation and inter-group classification

Over 600 metabolites were identified from serum samples through a widely targeted metabolomics approach. We used OPLS-DA to identify differences between the control and patient groups. The OPLS-DA score plot showed that the two groups have a tendency to cluster within groups and separate between groups (Fig. 1).

**Fig. 2:**
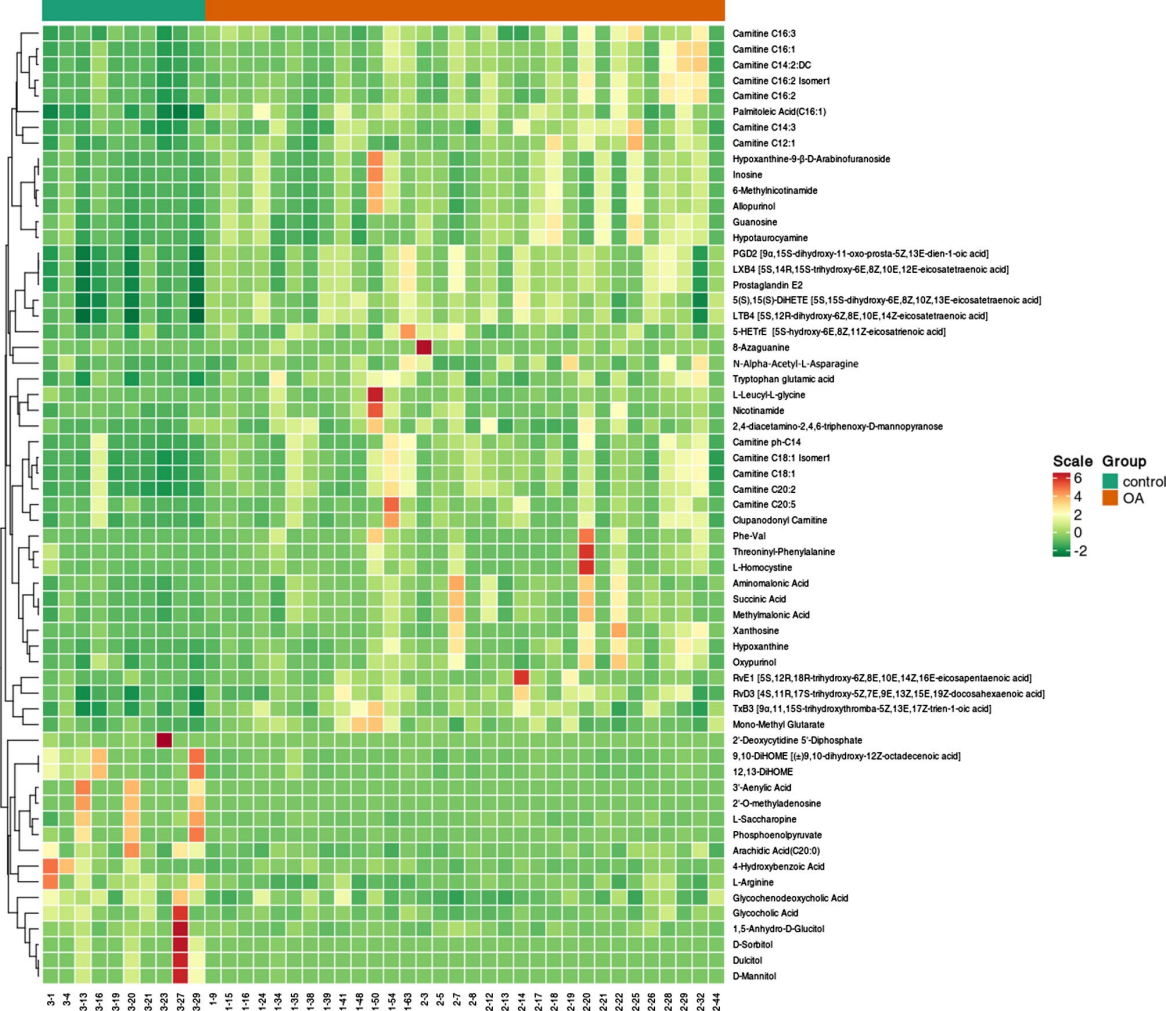
Heatmap presentation of 61 differential metabolites in the KOA group versus the control group.The horizontal axis shows 42 participants and the vertical axis shows 61 different metabolites. The numbers beginning with 3 are the control group, and the numbers beginning with 1 and 2 are the patient group

**Fig. 3:**
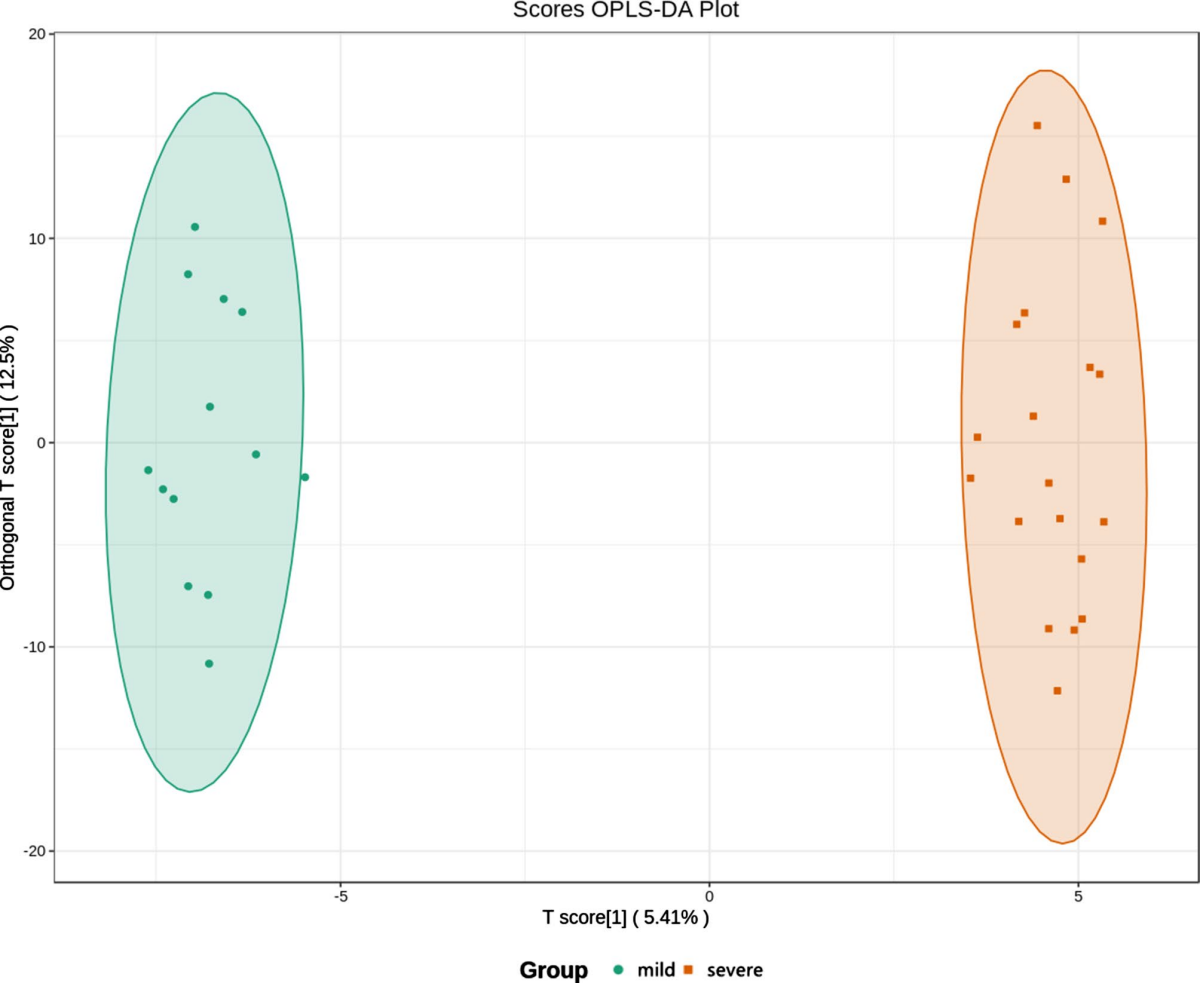
Plot of OPLS-DA scores for the mild versus severe KOA groups. The circles represent patients with mild KOA and the squares represent patients with severe KOA. The two groups had obvious trends of intra-group aggregation and inter-group separation

Unlike healthy controls, the patient group had 61 differentially expressed metabolites (45 upregulated and 16 downregulated) in the serum (Fig. 2). Differential metabolites that were upregulated included palmitoleic acid (C16:1), carnitine C20:2, hypoxanthine, xanthosine, and N-α-acetyl-L-asparagine, which were positively correlated with KOA. The same method was chosen to analyze mild and severe KOA to obtain OPLS-DA score plots and differential metabolites. There was a clear trend of separation between the mild and severe KOA groups (Fig. 3).

**Fig. 4:**
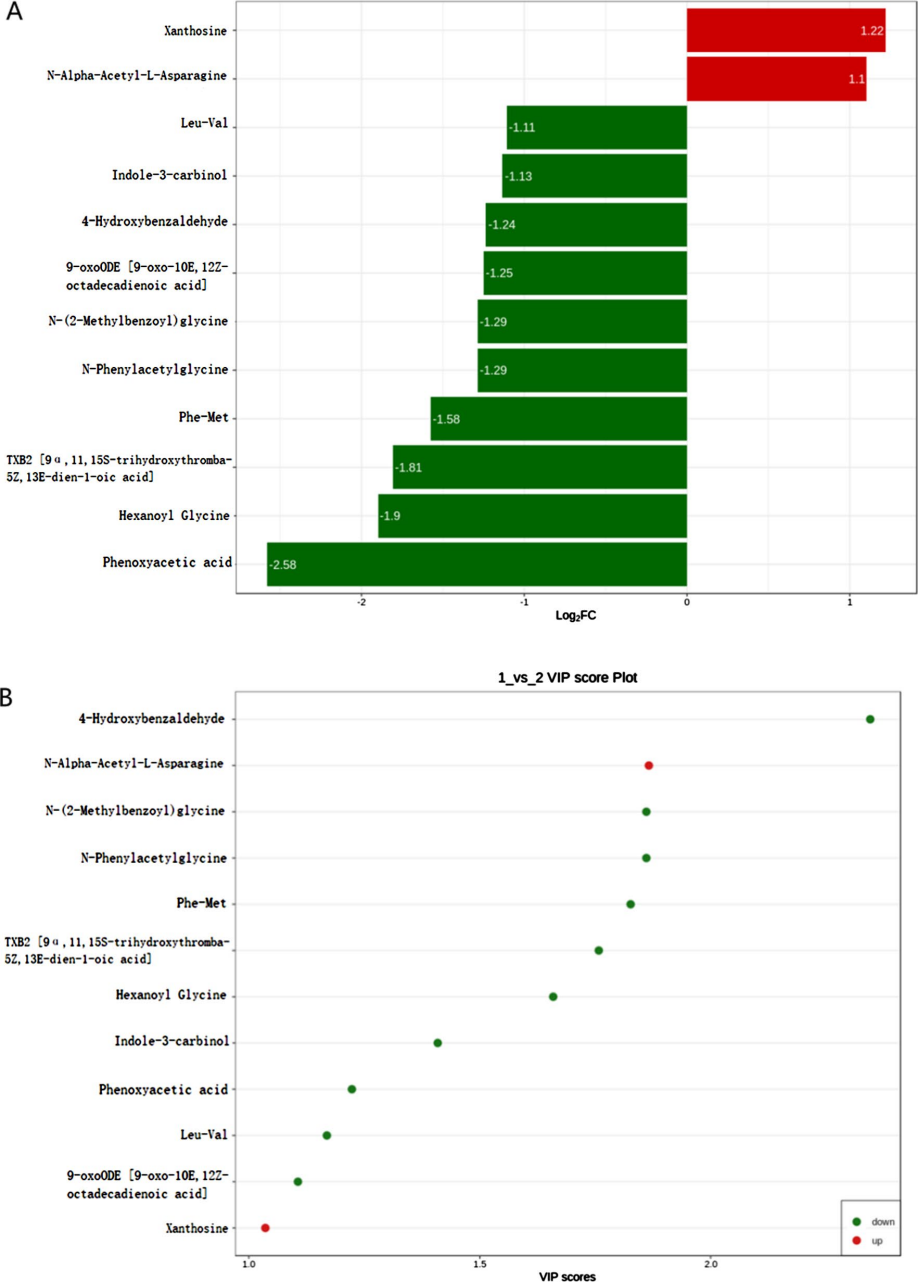
Identification of differentially expressed metabolites between mild and severe KOA. Xanthosine and N–acetyl-L-asparagine are metabolites associated with severe KOA. (**A**) The 12 differentially expressed metabolites ranked by log2 fold change (Log2 FC). (**B**) The 12 differentially expressed metabolites ranked by VIP

**Fig. 5:**
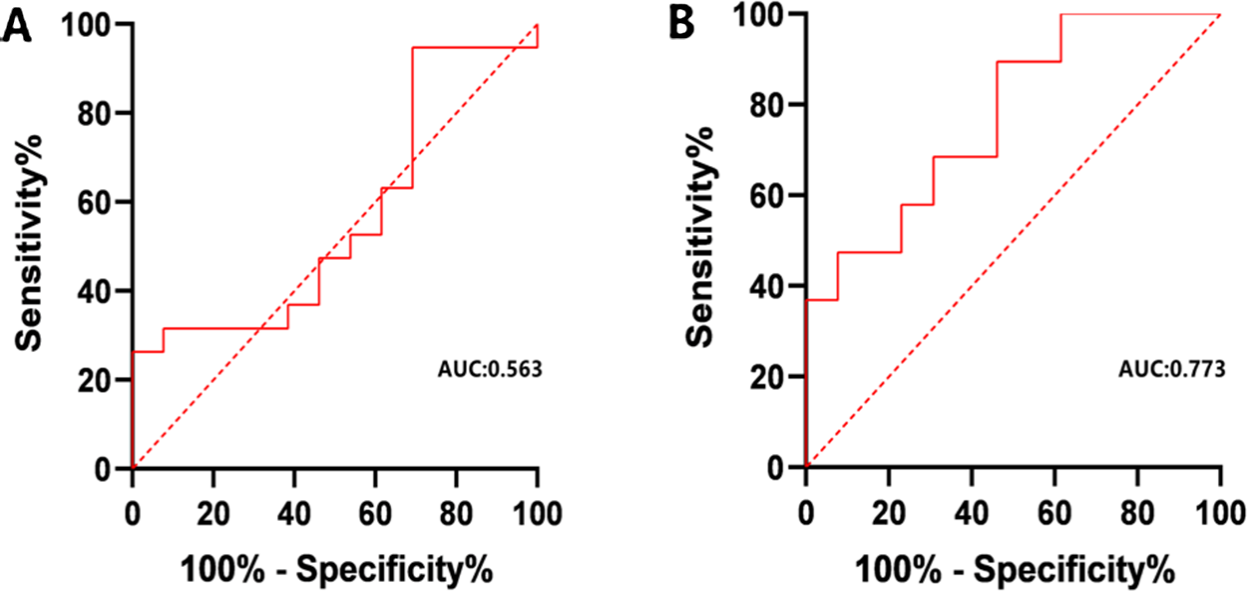
ROC curve analysis for the differential metabolites: xanthosine (**A**) and N-α-acetyl-L-asparagine (**B**). The area under the ROC curve for xanthosine is 0.563, however N–acetyl-L-asparagine is 0.773

Patients with severe KOA had 12 differentially expressed metabolites in the serum (Fig. 4). Two metabolites were positively correlated with KOA severity, xanthosine and N-α-acetyl-L-asparagine. Ten metabolites were negatively correlated with KOA severity, including hexanoyl glycine, N-phenylacetylglycine, and 4-hydroxybenzaldehyde. Therefore, xanthosine and N-α-acetyl-L-asparagine may be key metabolites to identify the severity of KOA. We assessed the feasibility of using xanthosine and N-α-acetyl-L-asparagine as potential predictors to differentiate the severity of KOA patients by ROC curve analysis. ROC curve analysis showed that N-α-acetyl-L-asparagine (AUC: 0.773) was a good predictor of late KOA (KL grade 3 and 4) (*P* < 0.05) (Fig. 5).

### Metabolic pathway enrichment analysis of differential metabolites

**Fig. 6:**
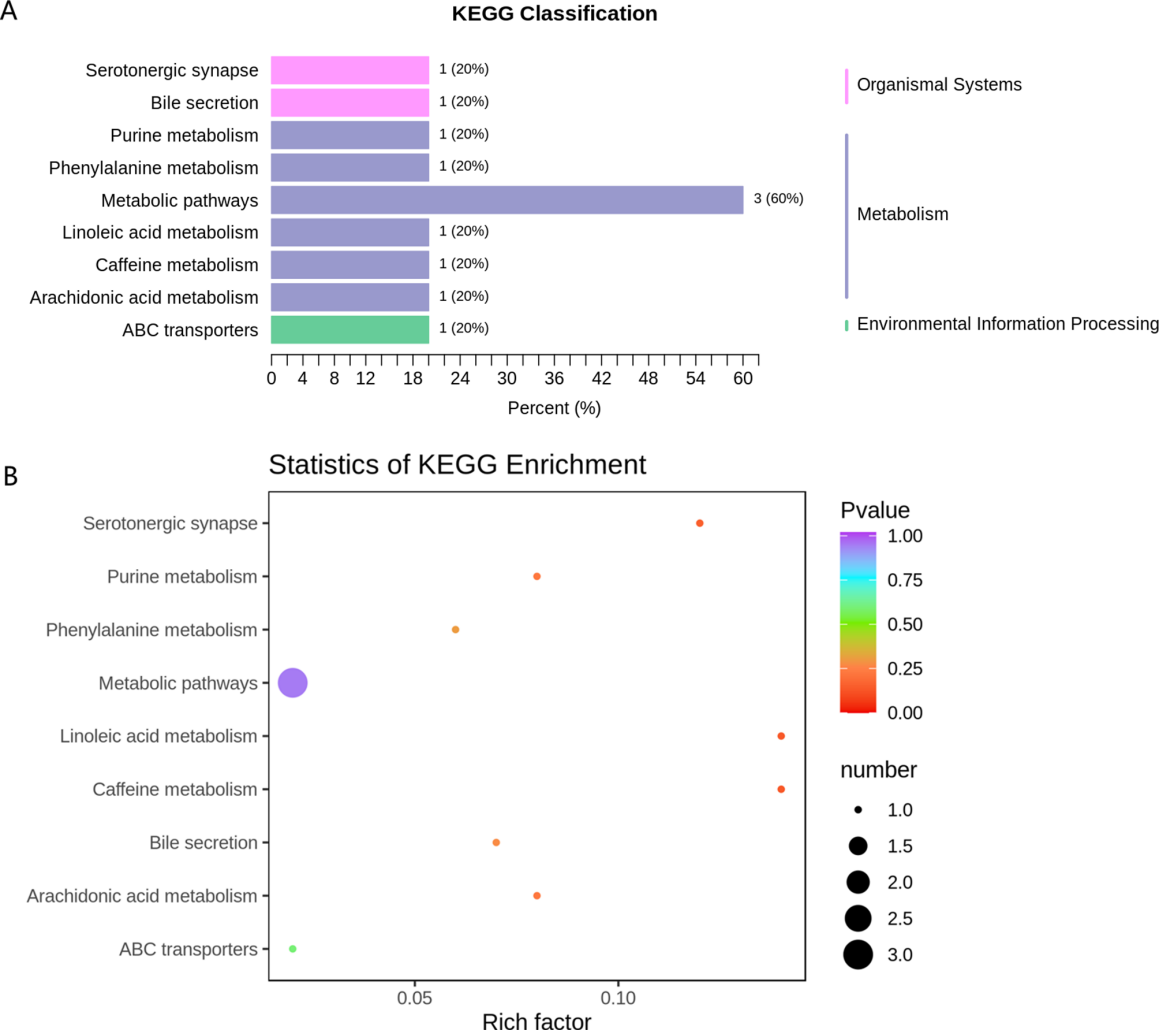
Pathway enrichment analysis of differentially expressed metabolites. (**A**) KEGG classification of differentially expressed metabolites between mild and severe KOA. (**B**) KEGG pathway enrichment analysis of differentially expressed metabolites

To investigate the effects of metabolites on metabolic pathways, pathway enrichment analysis was performed using the KEGG database to elucidate the role of the differentially expressed metabolites in the progression of KOA [26–28]. The KEGG annotation results for the mild and severe KOA groups were classified according to the pathway type and divided into 3 categories: organismal systems, metabolism, and environmental information processing (Fig. 6A). The enriched pathways were mainly associated with linoleic acid, caffeine, serotonergic synapse, purine, and arachidonic acid metabolism (Fig. 6B).These pathways need to be further validated in future studies for their relevance to severe knee osteoarthritis.

## Discussion

This study completed a metabolomic analysis of KOA patients in southern China using widely targeted metabolomics. In the first part, serum samples were collected from the patient and control groups to identify KOA-related metabolic markers by metabolomic analysis. In the second part, we distinguished between patients with mild and severe KOA according to the KL grading scale, obtained key biomarkers associated with KOA severity, and explored metabolic pathways for these biomarkers. A total of 45 metabolites were associated with KOA and were potential biomarkers of KOA. Xanthosine and N-α-acetyl-L-asparagine were the key metabolites associated with radiological severity. According to ROC curve analysis, metabolite N-α-acetyl-L-asparagine was highly correlated with severe KOA. High N-α-acetyl-L-asparagine levels may be a key biomarker for identifying KOA patients at high risk of progression.

Metabolomics is a young omics technology that identifies and quantifies metabolites from a variety of tissues [17]. Liquid chromatography–MS or gas chromatography–MS and nuclear magnetic resonance spectroscopy are the common analytical methods used in metabolomics [29]. Metabolomics has been widely applied to detect biomarkers of OA [30, 31]. Synovial fluid [30], cartilage [32], serum and plasma [33] are frequently used samples for metabolomics testing. Peripheral blood samples are often the first choice for metabolomic studies because of their availability and ease of monitoring the overall metabolic profile of the body [34].

N-α-acetyl-L-asparagine was identified as a key biomarker that could identify patients with severe KOA. The discovery of this metabolite, which is highly correlated with severe KOA, was a novel finding. N-α-acetyl-L-asparagine is an amino acid metabolite, but it has hardly been reported in the literature. Perturbations in amino acid metabolism are possibly closely related to the development of OA [35]. Leucine, isoleucine and valine are essential amino acids and are collectively referred to as branched-chain amino acids (BCAAs). Studies have shown that the occurrence of OA is associated with dysregulated concentrations of BCAAs [31]. The ratio of BCAAs to histidine is also considered as a biomarker of KOA [19]. Arginine is a semi-essential amino acid for humans [36]. Zhang et al. [37] found a significant decrease in arginine in KOA patients, probably due to excessive arginine catabolism. Werdyani et al. [38] verified the correlation between KOA and arginine deficiency by plasma metabolomics analysis of KOA patients. Alanine is associated with subchondral osteosclerosis in OA [39] and is also considered an important biomarker for identifying patients with OA [40]. Amino acids can be used for diagnosis and treatment of OA. Jiang et al. [41] found that mice in the OA model group had inhibition of exosomes and increased apoptosis, and after intervention, the glutamine metabolism level of chondrocytes increased, motility and cell function improved, and OA was relieved. OA is now considered an inflammatory disease [42], and glutamine has been found to reduce the inflammatory response of chondrocytes by inhibiting nuclear factor-κB activity, thus exerting a therapeutic effect [43]. Amino acid metabolism has become a current research hotspot due to the important role it plays in the occurrence, development, diagnosis and treatment of OA. Our study confirmed this. N-α-acetyl-L-asparagine is an amino acid metabolite, a novel biomarker for KOA, which helps to identify patients with severe KOA. However, there are few studies on N-α-acetyl-L-asparagine, and further research is needed to explore the role of N-α-acetyl-L-asparagine in the occurrence and development of KOA.

Differential metabolite enrichment analysis of severe versus mild knee osteoarthritis involves multiple pathways including arachidonic acid metabolism and linoleic acid metabolism. OA has long been regarded as a degenerative disease of cartilage. In recent years, as research into the pathogenesis of OA has intensified, there is increasing evidence that OA is an inflammatory disease [44].KOA patients often suffer from synovitis, which is considered one of the causes of joint pain [45, 46]. Synovial inflammation is accompanied by the release of pro-inflammatory mediators such as prostaglandin E2 (PGE2), nitric oxide (NO) [47, 48].Study finds arachidonic acid metabolism-related genes strongly associated with synovitis [49]. In clinical practice, patients with severe KOA are often associated with more severe joint pain than those with mild KOA. This may be due to the fact that arachidonic acid metabolism affects synovitis, which in turn leads to the production of more inflammatory factors, allowing patients to experience more severe knee pain.This phenomenon is consistent with the findings of our study. However, we did not further test the PGE2 levels of the patients, and we will delve into this mechanism in future studies.

There were some limitations to this study. First, it was a single-center study with a small sample size. In the future, more patients need to be included to validate the findings. Second, dietary habits may have some effect on the metabolomics of blood samples [50], and this study failed to strictly standardize this point. Third, the study failed to measure patients’ levels of PGE2. Fourth, most pathways in pathway enrichment analysis involve fewer metabolites, so the accuracy of pathways predicted is not high.

## Conclusions

KOA is a common disease in the middle-aged and elderly populations, and an increasing number of people are experiencing the effects of this disease in the context of an aging population. Metabolomics is a new histological approach. In this study, we applied a broadly targeted metabolomics approach to investigate the metabolite profile associated with the occurrence of KOA. We revealed that high N-α-acetyl-L-asparagine levels are a key biomarker for identifying severe KOA, which was a novel finding. The present study revealed the relationship between amino acid metabolism disorders and KOA, but further in-depth studies are needed to analyze the relationship between N-α-acetyl-L-asparagine and KOA. Arachidonic acid metabolism is more active in patients with severe KOA.

## Data Availability

The datasets generated and/or analyzed during the current study are not publicly available due the confidentiality of the participants’ data but are available from the corresponding author on reasonable reques.

## Abbreviations

KOA: knee osteoarthritis
KL: Kellgren–Lawrence
OA: osteoarthritis
BMI: body mass index
UPLC: ultra-performance liquid chromatography
MS: mass spectrometry
OPLS-DA: orthogonal partial least square-discriminate analysis
VIP: Variable importance in projection
ROC: Receiver operating characteristic
AUC: area under the curve
KEGG: Kyoto Encyclopedia of Genes and Genomes
BCAAs: branched-chain amino acids
